# Supplementary material for: In vitro and in vivo evaluation of Ulva lactuca for wound healing
Source: PLoS One. 2025 Jan 8;20(1):e0311037. doi: 10.1371/journal.pone.0311037 (PMC11709284; doi:10.1371/journal.pone.0311037)
Supplement: Attachment — Submitted filename: Response_to_Reviewers.docx [file pone.0311037.s001.docx]

**Responses to the Reviewers**

Reviewer #1: The manuscript is about investigating wound healing, antioxidation, anti-inflation and cell profanation. There are a suite of tools that they used to reach their conclusion. Although the authors claimed that there is a lack in the information about the role of Ulva, they mentioned several similar studies in the Discussion. They should mention exactly in which ways their work is different

1- Line 34: should start with a capital letter

**Response:** Thank you sincerely for your meticulous review and valuable feedback. We have made the corresponding necessary modification accordingly.

2- why did the authors used the phosphate-buffered saline solution instead of the ointments used normally for the injury to compare the effect with what is used normally?

**Response:** Initially, during *in vitro* testing of the biological functions of *Ulva* extract, it was dissolved in water. For subsequent animal experiments, we used PBS solution instead of ointments, as PBS is readily available in the laboratory and easy to prepare.

3- it would be useful to have information about using the combination of the two extracts

**Response:** We have added a corresponding description to the Discussion section accordingly.

4- several sentences in the introduction part need references to validate them, especially the first 3 sentences, and the line 70-73

**Response:** We have added a total of 6 references [1,10-14] accordingly.

5- In Material and Methods: Please mention the type of the flask the seaweed powder and the ethanol were put in, because at 60 °C ethanol will evaporate. Also mention the concentration of the ethanol used

**Response:** We have added a corresponding description to the Materials and Methods section.

6- line 167: remove the extra word “ethanol”

**Response:** We have removed the extra “ethanol” accordingly.

7- please justify the use of only female mice

**Response:** *Ulva* extracts are not hormonal substances, and the treatment is applied topically to the animals’ skin. Therefore, we believe the efficacy of *Ulva* extracts is not influenced by the animals’ sex. Female mice were chosen for this study simply because they are more readily available to purchase.

8- Results: line 203: add “extract” after the aqueous

**Response:** We have added “extract” after the term “aqueous.”

9- In the caption of figure 1, please add the number of replicates

**Response:** We have added the number of replicates accordingly.

10- Please add the scale for the bar on the images

**Response:** We used [Adobe® Photoshop®](https://www.adobe.com/tw/products/photoshop/landpa.html) software to enhance the resolution of our graphics and improve the clarity of the scale bars.

11- Line 257-260: should be in the materials and the methods

**Response:** We have relocated the corresponding text to the Materials and Methods section accordingly.

12- It would be more informative if the photos were analyzed using image processing tool to validate the measurements

**Response:** We have added an explanation of the image processing tool in the Materials and Methods section under the “Animals and mouse wound healing model” subsection.

13- The Discussion section needs more information to elaborate further on the possible causes that enhanced the recovery. Which are the potential active compounds that could contribute to the healing?

**Response:** In response to your valuable comment and based on Reviewer 1’s suggestion, we have added an explanation of the components and bioactivity of *U. lactuca* to the Introduction section to clarify the potential factors contributing to enhanced recovery.

14- The conclusion part needs to be reformulated and should be to the point giving the take-home message, and not repeating the results.

**Response:** We rewrote the Conclusion accordingly.

Reviewer #2: This study investigates the effects of the seaweed Ulva lactuca (U. lactuca) extracts on wound healing, encompassing both in vitro and in vivo experiments. The research topic is novel and holds practical value. The experimental design is mostly sound, and the data analysis methods are appropriate. However, several issues require further improvements.

1. The introduction lacks sufficient background information on U. lactuca. It is recommended to supplement it with more extensive literature reviews on its components and bioactivity to provide a stronger theoretical basis for the study.

**Response:** We have added an explanation of the components and bioactivity of *U. lactuca* to the Introduction section to clarify the potential factors contributing to enhanced recovery.

2. In the results section, the statistical analysis for Figure 2 should include significance indicators in the figure legends. The results of the cell migration assay in Figure 4 should be quantitatively analyzed, providing data on migration rates.

**Response:** We have added the statistical analysis for Figure 2 and included a quantitative analysis for Figure 4. The formula for the scratch closure rate is now provided in the Materials and Methods section under the “*In vitro* migration/wound healing assay” subsection.

3. It is suggested that the discussion section compares the findings of this study with other research on seaweed-promoted wound healing, highlighting similarities and differences.

**Response:** We have compared the findings of our study with those of other reports on seaweed-promoted wound healing in the Discussion section.

Ultimately, we would like to express our sincere gratitude to the editors and reviewers for their positive and constructive criticism. The manuscript has vastly benefited from your valuable and insightful comments and suggestions. We look forward to hearing from you and would be happy to address any further concerns, if required. We hope this further pushes the manuscript closer to publication in your esteemed journal.
